# “French Phage Network”—Third Meeting Report

**DOI:** 10.3390/v10030123

**Published:** 2018-03-10

**Authors:** Mireille Ansaldi, Laurent Debarbieux, Sylvain Gandon, Marie-Agnès Petit, Paulo Tavares, Pascale Boulanger

**Affiliations:** 1 Laboratoire de Chimie Bactérienne, UMR 7283 CNRS, Aix-Marseille Université, Institut de Microbiologie de la Méditerranée, 13402 Marseille, France; 2 Department of Microbiology, Institut Pasteur, 75015 Paris, France; laurent.debarbieux@pasteur.fr; 3 Centre d’Ecologie Fonctionnelle et Evolutive (CEFE)—UMR 5175, CNRS, Université de Montpellier, 34293 Montpellier, France; sylvain.gandon@cefe.cnrs.fr; 4 Micalis Institute, INRA, AgroParisTech, Université Paris-Saclay, 78350 Jouy-en-Josas, France; marie-agnes.petit@inra.fr; 5 Institute for Integrative Biology of the Cell (I2BC), CEA, CNRS, Université Paris-Sud, Université Paris-Saclay, 91190 Gif-sur-Yvette CEDEX, France; paulo.tavares@i2bc.paris-saclay.fr

**Keywords:** bacteriophage, bacteria, infection, co-evolution, virulence, resistance, phage therapy, structural biology, genomics, France

## Abstract

In its third year of existence, the French Phage Network (Phages.fr) is pursuing its expansion. With more than 25 groups, mostly based in France, working on the various aspects of phage research, the network has increased its visibility, interactivity, and activity. The third meeting of the Phages.fr network, held on November 2017 at the Gif-sur-Yvette Centre National de la Recherche Scientifique (CNRS) campus, was a great opportunity for many young scientists to present their work and interact with more senior scientists, amongst which several were invited from abroad. Here we provide a summary of the work presented at this occasion during the oral presentations and poster sessions.

## 1. Introduction

This year’s meeting was held 100 years after the publication of Felix d’Hérelle’s seminal paper in 1917 that proposed the name of bacteriophage for the filterable agent capable of lysing bacteria, recognizing its potential to eradicate bacterial pathogens [1]. It is amazing to see how much bacteriophages have recently been in the news, in scientific journals as well as in the general public-oriented press. Important discoveries were published in 2017, such as the way phages communicate with each other [2], the mechanism used by phages to cross the epithelial cell layers [3], the discovery of a totally new class of non-tailed phages in the oceans [4], and the identification of over 264,000 uncultured phage genomes recovered from many different environments [5]. Moreover, recent cases of successful phage therapy have created a momentum in the context of an increasing public health threat posed by antibiotic-resistant bacteria [6].

Phage research is by essence interdisciplinary, focusing on very diverse aspects of phage biology spanning from basic mechanisms and ecology to biotechnology and bacterial diseases therapy. The French Network is an excellent illustration of this interdisciplinarity, providing a solid base to foster collaborative research among its members and with the rapidly growing community interested in bacteriophages. More information on the network can be found on the dedicated website, Phages.fr, that was presented during the meeting by Rémy Froissart (CNRS, IRD, Université de Montpellier).

**Figure 1:**
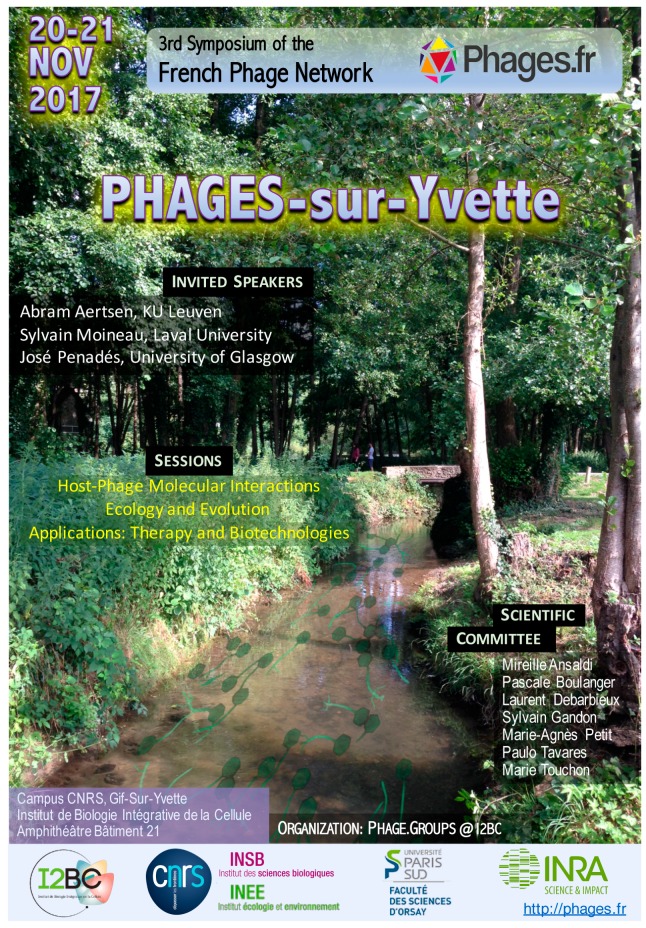
Poster of the third meeting of the French Phage Network.

After the two first meetings in Montpellier (2015) and Marseille (2016), Pascale Boulanger from the Institute for Integrative Biology of the Cell (I2BC) organized the third meeting of the French Phage Network in November 2017 on the Gif-sur-Yvette campus (Figure 1). This “Phages-sur-Yvette” conference brought together more than 100 researchers coming from all regions in France as well as several colleagues from the UK, Switzerland, Belgium, and Canada. This year the invited speakers were: Sylvain Moineau (Université de Laval), Abram Aertsen (KU Leuven), and José Penadés (University of Glasgow). The meeting allowed many interactions to take place between young and consolidated scientists from academia as well as from the private sector and from hospitals.

In this conference report we present an overview of oral presentations given at the meeting. The list of posters presented is compiled in Table 1. The meeting was organized into three sessions: (i) Host-Phage Molecular Interactions; (ii) Ecology and Evolution; and (iii) Therapy and Biotechnology.

## 2. Scientific Sessions

### 2.1. Host-Phage Molecular Interactions

The session started with a lecture by Sylvain Moineau (Université de Laval, Canada) entitled “Exploiting CRISPR-Cas systems to study phage biology”. Indeed, one has to remember that CRISPR-Cas systems were not designed to engineer (eukaryotic) genomes but are a bacterial immunity system to protect against phage infection and foreign DNA [7]. As phages evolve faster than bacteria, they are able to fight back and anti-resistance systems have been recently described, such as the anti-Cas9 system that can be used as a tool to control Cas9 activity [8]. This talk also stressed that the CRISPR field provides useful tools to study bacteriophages and engineer virulent phage genomes, which can additionally be used for undergraduate education training [9,10].

Moira Dion’s (Université de Laval) talk also dealt with CRISPR as part of a systematic microbiological analysis of a cohort of 700 children and their mothers. The CRISPR regions of a large collection of *Escherichia coli* strains isolated at three time points from the feces samples of this cohort were systematically sequenced and analyzed. Interestingly, no increase in the global array length was observed with time, and no correlation was found between phage sensitivity and the composition of the CRISPR arrays. Using the same cohort, Aurélie Mathieu (MICALIS, INRA) designed a pipeline to identify *E. coli* strains that contain active prophages. Her first conclusions indicate that the majority of *E. coli* isolates from the cohort are lysogens and that most strains are resistant to a set of 80 temperate phages isolated from the same strain collection.

The two following talks addressed the question of how phage-encoded proteins could modify the host physiology under non-lytic conditions. Aurélia Battesti (CNRS, Aix-Marseille Université) showed that the prophage-encoded regulator AppY bears two distinct regulatory functions: it acts as a stabilizer of the general stress response sigma factor RpoS and as a transcriptional regulator per se, modifying the ability of the *E. coli* host motility. Laurent Debarbieux (Institut Pasteur, IP) described a bacterial 2-hybrid screen to identify molecular partners of gp92 encoded by a virulent phages infecting *Pseudomonas aeruginosa* [11]. Interestingly, gp92 was shown to interact with MucA, an anti-sigma factor involved in the *P. aeruginosa* mucoid phenotype.

Three talks then focused on various aspects of phage T5 biology, delivering novel information on the infectious cycle of this model phage and revealing a fertile ground for new discoveries. In the first talk, Cécile Breyton (Institut de Biologie Structurale, CNRS, CEA) described the structure of the T5 tail tube. This important work suggests that Siphoviridae use a conserved mechanism to trigger DNA delivery into their host using the tail tube proteins as a scaffold, whereas the tape measure protein would be involved in signal transduction allowing DNA ejection [12]. Ombeline Rossier (I2BC, Université Paris-Saclay) then related the cellular impacts of the essential nuclease A1 that set the basis for cellular biology studies of this virulent phage. With the objective to engineer mutants in the strictly virulent phage T5, Luis Ramirez (I2BC, Université Paris-Saclay) adapted CRISPR-Cas9 technology to improve the screening of T5 mutants engineered by homologous recombination. He determined that two of the highly conserved T5 genes were non-essential for the T5 infectious cycle.

The second part of the Host-Phage Molecular Interactions session started with Abram Aertsen (KU Leuven), who developed the concept of “farming phages”. Based on previous results of studies using the P22/*Salmonella* Typhimurium model [13,14], the idea emerged that a phage carrier state allows host farming by keeping the naïve hosts that are immune to infection for a while, and then return to being sensitive to infection, providing new “grass” for the phage to prey on.

Marc Monot then described the PhageTerm tool, which allows a fast and accurate determination of the packaging mechanism (*cos* versus headful). This tool brilliantly exploits new generation sequencing (NGS) read number biases observed at natural phage DNA termini [15]. On the same topic of phage DNA packaging, Leonor Oliveira described the complex process of terminase assembly and DNA target recognition in the model SPP1/*Bacillus subtilis*. Interestingly, the large terminase domain TerL binds and cuts DNA very specifically and at a very precise position, although the DNA sequence by itself is not important and can be degenerated [16].

### 2.2. Ecology and Evolution

José Penadés (University of Glasgow) discussed the fascinating biology of phage-inducible chromosomal islands (PICIs). He showed that, although identified in Gram-positive organisms, PICIs are ubiquitous and parasite a wide range of hosts and prophages. Interestingly, whereas the induction mechanism of PICIs found in Gram-positive genomes is based on the inactivation of a repressor, it involves an activator in Gram-negative organisms [17,18].

The next talk by Marta Mansos Lourenco (IP) asked the question of the fate of virulent bacteriophages in the gastrointestinal tract (GIT) that do not always replicate efficiently. The approach used involves a systematic RNA-sequencing of an *E. coli* host strain in various sections of the GIT and several host genes were identified as modulators of phage replication. In vivo studies of bacteria-phages interactions are complex and certainly require modeling, which is the objective of the work presented by Jorge Sousa (IP). An ecological and evolutionary individual-based model was designed that will be helpful to better understand such complex relationships.

Olivier Schiettekatte (IP) described the isolation, genome sequencing, and characterization of three novel myoviruses that infect the bacterial pathogen *Leptospira* sp., highlighting the presence of numerous phage genes of unknown function. Nathalie Van Der Mee-Marquet (CHU Tours) reported studies of prophages in human isolates of *Streptococcus agalactiae*. Sequencing of a subset of the isolates genomes revealed the presence of numerous prophages whose role in bacterial pathogenesis was discussed.

### 2.3. Therapy and Biotechnology

In her talk, Cindy Fevre (Pherecydes Pharma) exposed a number of recent clinical cases that involved the compassionate use of phage therapy in France. Thanks to the good manufacturing practices (GMP)-quality phages provided by the company, several patients from the Hospices Civils de Lyon were treated in 2017, broadening our knowledge of the practices and side effects of this reviving therapy. Harald Brüssow (KU Leuven), who was involved in several clinical trials during his research career at Nestlé, alerted the community on the limitations of the reductionist principle [19,20]. According to his experience, the success of future phage therapy trials relies on both the extension of the reductionist approach to more complex mice models in research labs and on the widespread implementation of exploratory clinical observations.

On a prospective side, Julien Lossouarn (MICALIS, INRA) presented a screen to isolate virulent phages active on vancomycin-resistant *Enterococcus faecium* clinical isolates. He described a preliminary characterization of several phages with distinct properties. Aiming at developing phage therapy for agricultural applications, Fernando Clavijo (CNRS, Aix-Marseille Université) investigated the prophage content of the emerging plant pathogen *Xylella fastidiosa*, now present in several European countries, as prophage abundance can prevent efficient infection by virulent phages. 

Another aspect of this session concerned the biotechnological applications of bacteriophages. Alfonso Jaramillo (University of Warwick) exposed several methods of host-phage engineering for the directed evolution of biomolecules. Directed evolution systems were developed using M13 (filamentous) and T7 (virulent) phage models [21,22]. Finally, Nathalie Garrec (Centre Scientifique et Technique du Bâtiment) described a feasibility study to use phages active against environmental isolates of *P. aeruginosa* to disinfect plumbing material. A long-term goal would be to apply the results obtained in a pilot-water plant to water distribution networks and obtain approval for phages as biocides.

## 3. Discussions and Perspectives

The 2017 meeting confirmed that the research activity in the French Phage network is intense and that a young generation of “phagists” is emerging. The friendly atmosphere of this meeting was instrumental to developing fruitful discussions during poster sessions, lunches, and dinners, as well as to initiating collaborations.

Besides this annual meeting, the network also organizes workshops addressing specific questions including Phage Bioinformatics, Phage Therapy, Ecology, and the Molecular Basis of Phage Infection, which are reported on the Phages.fr website. As stated before, phage research is necessarily pluridisciplinary. Such meetings and workshops are key opportunities for researchers, who each work on a given model by using specific approaches, to address new questions and expand their perspectives on this model. Our network reflects the large diversity of biological and applied questions related to the study of bacterial viruses. Members of the network come from different origins (academic, private, medical) and their diverse expertise is at the heart of rich exchanges. Everyone brings their knowledge, shares it, and leaves with implemented ideas and suggestions to progress in his/her own research activity, for the benefit of all.

We would like to encourage people interested in our network to have a look at our website for future activities. The next annual meeting is scheduled to occur in the fall of 2018 at the Université de Bordeaux. More information on forthcoming workshops will soon be available on our webpage. 

## Figures and Tables

**Table 1:** Posters presented during the Phage-sur-Yvette 2017 meeting.

Poster Title	Presenter (Group)	Session
Unraveling the dependence of SPP1 bacteriophage on molecular chaperones from the host *Bacillus subtilis*	Lia Marques Godinho (P. Tavares)	Host-Phage Molecular Interactions
Nitric oxide-driven prophage maintenance involves unsuspected activity of NorV(W) reductase	Stéphanie Champ (M. Ansaldi)	
Control and maintenance of prophages in *Salmonella enterica*	Astrid Wahl (M. Ansaldi)	
New insights into A1, a multitasking pre-early protein of bacteriophage T5	Léo Zangelmi (P. Boulanger)	
Sak4 of phage HK620 is an SSB-stimulated annealase that is involved in the lytic development of the phage	François Lecointe (M.A. Petit)	
Hidden phage Vp16 PDF features are essential for deformylase activity	Francesco Lavecchia	
Deciphering phage trajectories with interferometric microscope	Martine Boccara	
Genome-wide CRISPRi screen reveals bacterial requirements for phage infection	François Rousset (D. Bikard)	
Enological environment as a source of a novel tectivirus	Cécile Philippe (C. Le Henaff)	Ecology and Evolution
Survey on prophages infecting *Weissella cibaria* and *Weissella confusa*	Coralie Coudray-Meunier	
Phages infecting *Leuconostoc mesenteroides* in dairy products	Coralie Coudray-Meunier	
PhagesCollect: an updated and functional collection of dairy bacteriophages	Sarah Chuzeville	
Genetic characterization of two siphoviridae targeting marine magnetotactic bacteria from the Mediterranean Sea	Nicolas Ginet (M. Ansaldi)	
Gut Phageome: how to extract and analyze phage DNA	Camille D’humières (E. Rocha)	
Phages application to control *Pseudomonas aeruginosa* contaminations from terminal water points of use	Vanessa Magin	Therapy and Biotechnology
Efficacy of coliphages to prevent chicken embryo mortality	Angélina Trotereau (C. Schouler)	

## References

[1] D’Herelle F. (1917). Sur un microbe invisible antagoniste des bacilles dysentériques. CR Acad. Sci. Paris.

[2] Erez Z., Steinberger-Levy I., Shamir M., Doron S., Stokar-Avihail A., Peleg Y., Melamed S., Leavitt A., Savidor A., Albeck S. (2017). Communication between viruses guides lysis-lysogeny decisions. Nature.

[3] Nguyen S., Baker K., Padman B.S., Patwa R., Dunstan R.A., Weston T.A., Schlosser K., Bailey B., Lithgow T., Lazarou M. (2017). Bacteriophage Transcytosis Provides a Mechanism To Cross Epithelial Cell Layers. mBio.

[4] Kauffman K.M., Hussain F.A., Yang J., Arevalo P., Brown J.M., Chang W.K., VanInsberghe D., Elsherbini J., Sharma R.S., Cutler M.B. (2018). A major lineage of non-tailed dsDNA viruses as unrecognized killers of marine bacteria. Nature.

[5] Paez-Espino D., Chen I.-M.A., Palaniappan K., Ratner A., Chu K., Szeto E., Pillay M., Huang J., Markowitz V.M., Nielsen T. (2017). IMG/VR: A database of cultured and uncultured DNA Viruses and retroviruses. Nucleic Acids Res..

[6] Schooley R.T., Biswas B., Gill J.J., Hernandez-Morales A., Lancaster J., Lessor L., Barr J.J., Reed S.L., Rohwer F., Benler S. (2017). Development and Use of Personalized Bacteriophage-Based Therapeutic Cocktails To Treat a Patient with a Disseminated Resistant Acinetobacter baumannii Infection. Antimicrob. Agents Chemother..

[7] Labrie S.J., Samson J.E., Moineau S. (2010). Bacteriophage resistance mechanisms. Nat. Rev. Microbiol..

[8] Hynes A.P., Rousseau G.M., Lemay M.-L., Horvath P., Romero D.A., Fremaux C., Moineau S. (2017). An anti-CRISPR from a virulent streptococcal phage inhibits *Streptococcus pyogenes* Cas9. Nat. Microbiol..

[9] Trudel L., Frenette M., Moineau S. (2017). CRISPR-Cas in the laboratory classroom. Nat. Microbiol..

[10] Lemay M.-L., Tremblay D.M., Moineau S. (2017). Genome Engineering of Virulent Lactococcal Phages Using CRISPR-Cas9. ACS Synth. Biol..

[11] Blasdel B.G., Chevallereau A., Monot M., Lavigne R., Debarbieux L. (2017). Comparative transcriptomics analyses reveal the conservation of an ancestral infectious strategy in two bacteriophage genera. ISME J..

[12] Arnaud C.-A., Effantin G., Vivès C., Engilberge S., Bacia M., Boulanger P., Girard E., Schoehn G., Breyton C. (2017). Bacteriophage T5 tail tube structure suggests a trigger mechanism for Siphoviridae DNA ejection. Nat. Commun..

[13] Cenens W., Mebrhatu M.T., Makumi A., Ceyssens P.-J., Lavigne R., van Houdt R., Taddei F., Aertsen A. (2013). Expression of a Novel P22 ORF an Gene Reveals the Phage Carrier State in *Salmonella Typhimurium*. PLoS Genet..

[14] Cenens W., Makumi A., Mebrhatu M.T., Lavigne R., Aertsen A. (2013). Phage-host interactions during pseudolysogeny: Lessons from the Pid/*dgo* interaction. Bacteriophage.

[15] Garneau J.R., Depardieu F., Fortier L.-C., Bikard D., Monot M. (2017). PhageTerm: A tool for fast and accurate determination of phage termini and packaging mechanism using next-generation sequencing data. Sci. Rep..

[16] Djacem K., Tavares P., Oliveira L. (2017). Bacteriophage SPP1 pac Cleavage: A Precise Cut without Sequence Specificity Requirement. J. Mol. Biol..

[17] Bowring J., Neamah M.M., Donderis J., Mir-Sanchis I., Alite C., Ciges-Tomas J.R., Maiques E., Medmedov I., Marina A., Penadés J.R. (2017). Pirating conserved phage mechanisms promotes promiscuous staphylococcal pathogenicity island transfer. eLife.

[18] Martínez-Rubio R., Quiles-Puchalt N., Martí M., Humphrey S., Ram G., Smyth D., Chen J., Novick R.P., Penadés J.R. (2017). Phage-inducible islands in the Gram-positive cocci. ISME J..

[19] Sarker S.A., Ahmed T., Brüssow H. (2017). Persistent diarrhea: A persistent infection with enteropathogens or a gut commensal dysbiosis?. Environ. Microbiol..

[20] Sarker S.A., Sultana S., Reuteler G., Moine D., Descombes P., Charton F., Bourdin G., McCallin S., Ngom-Bru C., Neville T. (2016). Oral Phage Therapy of Acute Bacterial Diarrhea With Two Coliphage Preparations: A Randomized Trial in Children From Bangladesh. EBioMedicine.

[21] Brödel A.K., Isalan M., Jaramillo A. (2018). Engineering of biomolecules by bacteriophage directed evolution. Curr. Opin. Biotechnol..

[22] Brödel A.K., Jaramillo A., Isalan M. (2017). Intracellular directed evolution of proteins from combinatorial libraries based on conditional phage replication. Nat. Protoc..

